# The Effect of Cone-Beam Computed Tomography (CBCT) Evaluation on Treatment Planning after Endodontic Instrument Fracture

**DOI:** 10.3390/ijerph19074088

**Published:** 2022-03-30

**Authors:** Konstantinos Kalogeropoulos, Alexandra Xiropotamou, Despina Koletsi, Giorgos N. Tzanetakis

**Affiliations:** 1 Private Practice Limited to Endodontics, 11521 Athens, Greece; info@athensendo.gr; 2 Private Dental Practice, 11521 Athens, Greece; antaxiropotamou@gmail.com; 3 Clinic of Orthodontics and Pediatric Dentistry, Center of Dental Medicine, University of Zurich, 8032 Zurich, Switzerland; 4 Department of Endodontics, School of Dentistry, National and Kapodistrian University of Athens, 11527 Athens, Greece; gtzanet@dent.uoa.gr

**Keywords:** cone-beam computed tomography, instrument fracture, treatment planning, decision making

## Abstract

Intracanal instrument fracture is a procedural iatrogenic event during endodontic treatment that may affect treatment planning and eventually treatment outcome. Cone Beam Computed Tomography (CBCT) has offered several advantages, especially in endodontic cases in which information from conventional periapical radiograph may not be adequate to allow a precise treatment planning decision and a subsequent appropriate management of the cases. The present study was firstly conducted to assess the effect of CBCT evaluation on the decision-making process after instrument fracture; secondly, to introduce a new clinical approach in cases with fractured instruments located in the mesial roots of mandibular and maxillary molars. The study design was observational. The sample comprised all cases of mandibular and maxillary molars where an instrument fracture had occurred in the mesial roots. Two qualified (National and Kapodistrian University of Athens, Greece) and experienced (more than fifteen years of daily practicing) endodontists evaluated all the cases. The initial treatment plan made by evaluating periapical radiographs of each case was compared to the final plan set after CBCT evaluation. A marginal homogeneity test for paired data was conducted to test the concordance of treatment planning with periapical radiographs versus CBCT. Multivariable logistic regression was structured to identify predictors of modification in treatment planning following CBCT assessment, and to record estimators for decision to remove, bypass or retain the fragment. The level of statistical significance was pre-specified at *p* < 0.05. Of a total 52 cases evaluated, change in treatment planning with conventional periapical radiograph as a reference, following evaluation of CBCT, was observed in more than half of the teeth. The difference was statistically significant (*p* < 0.001). Apical location of the fragment was more likely to induce a perceived change in treatment planning after CBCT evaluation (*p* < 0.01). Canal merging induced 95% lower odds (*p* = 0.01) for taking a decision to remove or bypass, revealing that retaining the fragment was by far a more likely decision. A significant impact of CBCT preoperative evaluation on treatment planning for the management of such cases was demonstrated. Apical location of the fragment and canal merging seem to influence the decision-making process.

## 1. Introduction

Intracanal instrument fracture is a procedural complication which may challenge clinicians and frustrate patients [1]. Finding a retained instrument is not an uncommon situation, which may jeopardize the mechanical instrumentation and chemical disinfection of root canals [1,2,3]. Instrument fracture most commonly occurs in molars, [2,4,5], while the most frequent localization of the fragment corresponds to the mesial roots of mandibular molars followed by the buccal canals of maxillary molars [2], especially within the apical third of the canals [4,5,6]. A potential explanation might be the complexity of root canal anatomy that characterises these roots [7,8,9].

The management of instrument fracture remains a controversial issue in current clinical practice [10]. Removal of the fragment is considered the most preferable option [1], while both removal and bypassing of the fragment are mentioned as successful approaches as they satisfy the main objectives of root canal treatment [1,2,11]. However, in some cases, the integrity of tooth anatomy and function may be compromised due to potential complications. A number of complications have been reported during management, namely, perforations, ledges, new instrument fractures and unnecessary or excess dentin removal [2,6,12]. Therefore, in certain cases, leaving the fragment in situ seems to be the most conservative alternative treatment approach [13]. However, the prognosis may be uncertain, especially if a periapical lesion is present. In roots with two canals, which are often merging apically, the prognosis may not be significantly affected. However, apical merging cannot be completely assessed by an initial periapical radiograph. Furthermore, surgical management may also be considered as a treatment option during the follow-up examination, when a lesion is persistent or increases in size.

Treatment planning comprises preoperative assessment on various factors such as strategic importance of the tooth, existence of periapical lesion, risk of complications, position of the fragment in the canal, difficulty of root canal anatomy and available armamentaria and resources [2,12,13]. According to the European Society of Endodontology (ESE) position statement [14], limited field of view cone-beam computed tomography (CBCT) should be considered as a diagnostic tool in cases where treatment complications have occurred. In essence, its selective and careful usage is recommended in high difficulty cases [15]. Based on the existing literature, CBCT has lately been reported as a valuable tool that might increase confidence of the practitioners in critical decision making [16,17,18,19,20]. The impact of CBCT on the management of cases with instrument fracture is limited to in vitro studies related to the detection of fragments in the presence of filling materials inside the root canals [21,22,23,24,25]. So far, only one in vitro study has examined the contribution of CBCT to decision making in cases with instrument fracture compared to periapical radiographs [26]. The authors concluded that CBCT evaluation led mostly to a decision of removing or bypassing the fractured fragment during periapical radiographic evaluation to a decision of leaving the fragment in situ [26]. Although intracanal instrument fracture has been widely considered as a potential treatment complication, no in vivo study has been performed so far to assess the impact of preoperative CBCT evaluation in informing decision making regarding the management of cases with instrument fracture. 

Therefore, the main purpose of the present study was firstly to assess the effect of CBCT examination in decision making prior to management of an instrument fracture, and secondly, to introduce a new clinical approach regarding the management of cases with fractured instruments located in the mesial roots of maxillary and mandibular molars, assisted by the use of CBCT.

## 2. Materials and Methods

The present study was conducted from 1 July 2019 to 30 June 2020 and was approved by the Human Research Ethics Committee of School of Dentistry, National and Kapodistrian University of Athens, Athens, Greece (451-16 December 2020). The study population consisted of all cases of mandibular and maxillary molars referred for further management where an instrument fracture had previously occurred. More specifically, the study focused on fractures that had occurred in the mesial roots of the molars. All patients were informed about the type and design of the research and signed an informed consent.

Two qualified and experienced (KK, GNT) endodontists, with more than fifteen years of daily practicing, evaluated all cases in duplicate. A calibration procedure of the two clinicians took place in advance in 30% of the samples. The calibration was a two-step procedure. First, it was performed for periapical radiographs and second, it was carried out for CBCT. Subsequently, assessment of radiographs was performed independently by both investigators for the entire sample. Any disagreement was resolved through discussion and consensus was reached between the clinicians. A third experienced evaluator, with more than twenty years of daily endodontic practice, facilitated the disagreement discussions.

### 2.1. Clinical and Initial Radiographic Examination

All patients had nonsignificant medical history. Inclusion and exclusion criteria were set for the participation of the patients in the study as follows:

Inclusion criteria:

1. Maxillary and mandibular molars with instrument fracture in the mesial root
2. Informed consent by patients who wish to participate in the study

Exclusion criteria:

1. Patients with a contributory severe medical history such as immunocompromised patients or patients with a history of radiation involving the jaws.

During the first appointment, dental history was obtained, and thorough clinical and radiographic examinations took place. Clinical examination involved visual inspection of the area around the tooth of interest for any pathologic signs, such as swelling or sinus tract, palpation and percussion tests. In addition, periodontal examination was conducted, such as tooth mobility and periodontal pocket inspection.

Initial radiographic examination included periapical radiographs in two different horizontal angles by using the parallel cone technique. More specifically, during the first appointment, the following clinical and radiographic parameters were recorded:

- Tooth/teeth of interest;
- Root and canal with instrument fracture;
- Location of the fragment (cervical, middle, middle-apical, apical third);
- Presence of periapical, lateral or furcation lesion;
- Presence of signs (sinus tract, swelling) and symptoms.

After completion of clinical and initial radiographic examination, an initial treatment plan was set, based on the information obtained by periapical radiographs. Initial treatment plan decision and respective criteria for this decision were blindly recorded by the evaluators.

### 2.2. CBCT Examination and Imaging Protocol

CBCT examination was performed for each case fifteen days after the initial radiographic examination. This was conducted in order to enhance the objectivity of the evaluators and the reliability of the decision treatment planning procedure, and it eliminated recall bias. Again, a treatment plan was established based on the information obtained by CBCT examination. Treatment plan decision after CBCT examination and respective criteria for this decision were also blindly recorded by the evaluators. 

All CBCT examinations took place at the same radiologic clinic using the same unit (Planmeca ProMax 3Ds, Helsinki, Finland). The imaging protocol was selected according to diagnostic task, clinical findings and patients’ history. The imaging protocol had the following exposure parameters: Voltage was fixed at 90 kVp, whereas current settings were calculated automatically by the X-ray unit and the software, according to patients’ physical characteristics, varied from 6.3 mA to 12.5 mA. Total examination time was 60 s with an exposure time of 15 s, since the X-ray unit uses a pulsed exposure. Isotropic voxels with a size of 0.15 mm for 50 × 80 mm field of view were used. Slice thickness was 1 mm or even less (0.5 mm), and reconstruction of the volume data was performed in axial, cross-sectional and longitudinal views.

### 2.3. Statistical Analysis

All data acquired before and after CBCT evaluation were collected and classified according to their category into a spreadsheet and processed for statistical analysis. Baseline characteristics of the sample were presented through descriptive statistics and contingency tables. Pearson chi-square or Fisher’s exact tests were applied as appropriate. A marginal homogeneity test for paired data (Stuart–Maxwell) was performed to detect concordance of treatment planning decision making, or otherwise, based on conventional periapical radiographic assessment or CBCT evaluation of the same sample.

Univariable and multivariable logistic regression was performed on two occasions. First, to identify predictor variables such as tooth and jaw, location of the fragment within the root canal (converted to binary: more cervically/apically), canal merging and presence of lesion for modification in treatment planning regarding intracanal instrument fracture following CBCT assessment. Second, to estimate the effect of tooth and jaw, location of fragment within the root canal (converted to binary: more cervically/apically), canal merging and presence of lesion on treatment decision to remove or bypass compared to retaining the fragment after CBCT evaluation. For both examined outcomes, predictors were inserted sequentially, one at a time, and retained in the final model if *p* < 0.10. Model fit was checked through Hosmer–Lemeshow’s goodness of fit test. The kappa value for inter-rater agreement was 0.89 [95% Confidence Interval (CI): 0.76–1.0], revealing almost perfect agreement.

The level of statistical significance was pre-specified at *p* < 0.05 (two-sided). Statistical analyses were performed with STATA version 15.1 software (Stata Corporation, College Station, TX, USA).

## 3. Results

The sample consisted of 52 patients contributing an equivalent number of teeth (molars) with a fractured instrument. There was a very slight preponderance of female patients in the sample (27/52; 51.9%) compared to males. The mean age of the patients was 45.0 (standard deviation, SD: 14.1), with a range of 21.0 to 73.0 years old. 

Mandibular molars were the most represented teeth (36/52; 69.2%), with the vast majority being first molars (31/36; 86.1%), and the corresponding most prevalent canals being the buccal canal of the mesial root (25/36; 69.4%), followed by the lingual canal of the same root (10/36; 27.8). Maxillary molars with fractured instruments were identified in 16 out of 52 cases (30.8%), with 14/16 being first molars (87.5%), while the instrument fragment was detected mostly in one of the two buccal canals of the mesial root (13/16; 81.3%). The most prevalent localization of the fragment was the apical third of the root canal (27/52; 51.9%), followed by the middle (13/52; 25%) and the middle-apical (10/52; 19.2%). Apical location of the fragment was also more likely to induce a perceived change in treatment planning after CBCT evaluation of the canal (*p*-value < 0.01). Canal merge was identified in 28/52 root canals (53.8%). Moreover, a lesion was present in 43 out of 52 cases (82.7%) and symptoms were also identified in 39/52 (75%) patients (Table 1).

Of the 52 cases of instrument fractures, there was change in treatment planning, with conventional periapical radiography as a reference, after evaluation of the CBCT in more than half of the teeth (29/52; 55.8%, Table 1). Treatment planning with the aid of CBCT actually coincided with the ultimate management of the canal with the fragment in all but two cases, where a decision to bypass the fragment was altered to either retaining or surgical management without bypass. Additionally, another four cases retained the fragment as planned after CBCT evaluation but were also supplemented with surgical management. Initial plan for bypass of the fractured instrument according to conventional periapical radiography was decided for 27 out of 52 cases, while this was changed to retaining the fragment without bypass in the majority of assessments (18/27; 66.7%) and removal of the fragment in another 5 out of 27 (18.5%) following final assessment with CBCT (Table 2). 

Moreover, the initial plan for instrument removal resulted in the modification of treatment decision after CBCT assessment, specifically to retaining the instrument in 4/25 cases (16%) and to bypass in another 2 (8.0%). The majority remained as planned for removal even after CBCT evaluation (19/25; 76%). There was strong evidence for statistically significant difference between treatment planning decisions with conventional periapical radiography and after CBCT assessment. The *p*-value for the marginal homogeneity test for paired data (Stuart–Maxwell) was *p* < 0.001 (Table 2).

According to the logistic regression model, when alteration of treatment planning from conventional periapical radiographic assessment to final CBCT evaluation was considered as the outcome of interest, more apical detection of the instrument fragment presented 11.31-times higher odds (95% CI: 3.07, 41.76; *p*-value < 0.001) of incurring this alteration compared to more cervical one (Table 3). 

The multivariable logistic regression for the effect of tooth, localization of fragment, canal merge or presence of lesion on treatment decision to remove or bypass the fragment compared to retaining within the root canal, without bypass and based on CBCT evaluation, revealed the following: there was strong evidence that more apical detection of the fragment presented 97% lower odds (95% CI: 0.003, 0.30; *p*-value = 0.003) for informing a decision to proceed with removal or bypass compared to retaining, after adjusting for canal merging and tooth. In addition, canal merging induced 95% lower odds (95% CI: 0.01, 0.52; *p*-value = 0.01) for a treatment decision to remove or bypass, conditional on tooth and location of the fragment, revealing that retaining of the fragment was by far more likely in such cases (Table 4).

## 4. Discussion

To our knowledge, the present investigation is the first clinical (in vivo) study which has intended to evaluate the impact of CBCT preoperative evaluation on treatment planning of cases with intracanal instrument fracture. Based on evidence regarding the prevalence of this complication, mesial roots of mandibular and maxillary molars were selected for assessment. In addition, the decision to selectively investigate this issue on mesial roots of molars was driven by anatomical considerations regarding root morphology, possible canal curvatures and overall more pronounced complexity of treatment planning, characterizing these roots. The main finding was the significant change in initial treatment planning based on the evaluation of digital periapical radiographs, after CBCT assessment and interpretation. This occurred in more than half of the cases that were assessed. The latter is in agreement with the results of previous studies, which concluded that CBCT evaluation may lead to treatment plan alterations in 45–62% of cases with a high degree of difficulty [16,19,27]. Two occasions seem to influence decision making overall when an intracanal instrument fracture has occurred. First, the location of the fragment and second, the apical canal merging.

It is well known that the more apical the fragment is, the more difficult its removal appears [6,11]. In addition, in such cases, the removal of dentin from root canal walls is expected to be excessive, thus possibly jeopardizing the structural integrity of the tooth [12,28,29]. This may be potentially prevented if canals are merging apically, suggesting that retention of the fragment will probably not affect treatment outcome. In essence, the option of retaining the fragment is not new, as has been reported in earlier studies for treatment planning decision in such cases [1,13]. The present study, however, documents for the first time this alternative and confirms that it could serve as a viable treatment option in approximately half of the cases. As such, this study has elucidated a clear reasoning for CBCT preoperative evaluation, when a fractured instrument is located apically within the root canal. Nevertheless, even when canals are separate and removal or bypass of the fragment is considered as treatment of choice, especially in cases with periapical lesions, CBCT may certainly assist in the evaluation of the degree of canal curvature beyond which the instrument has fractured, thus leading to a more complete mapping of the possibilities for a successful removal. 

An additional important issue is canal merging apically. According to micro-CT studies, this feature is found in more than two-thirds of the mesiobuccal roots of first maxillary molars [9], whereas in mandibular molars, this accounts for a third of the cases [30]. However, the clinical element of apical canal merging cannot be accurately assessed from initial periapical radiographs and only speculations may be made by the clinician. This assessment may still prove difficult through CBCT evaluation, in cases where an inappropriate scan is selected for this purpose. However, CBCT is the sole diagnostic tool to conclude preoperatively whether the canals do merge or not. This pinpoints the central role of diagnosis in high-difficulty cases, leaving technical issues aside. So far, most investigators have focused on the study of technical aspects of the root canal treatment with a fractured instrument per se [6,11,12], while no assessment has been made on the justification of these attempts based on reliable diagnostic tools and according to the unique characteristics of each case.

A further interesting finding was related to the presence of a lesion and its effect on treatment planning, which was not found to be significant, even when a preoperative CBCT evaluation had taken place. This is substantially important if one considers that the majority of clinical actions, for the time being, are based on the presence, or not, of periapical lesions. During initial radiographic examination, no treatment plan of retaining the fragment without bypassing it was ultimately decided, although this treatment option was available for the evaluators. The evaluators were asked about that choice and the following clarification was provided: in cases where a lesion was present and the canals were separate, then the prognosis of the case would be poor. However, they could not be confident about whether the canals were merging or not. In addition, according to the dental history received during the initial examination, all remaining cases without lesion had proceeded to the referral dentist with symptoms of end-stage irreversible pulpitis (relief with cold stimulus), which translates to the presence of bacterial load, already by the time of initial management. Moreover, the evaluators were unaware of whether the referral dentist had used a rubber dam isolation during the initial access of the tooth. However, all the procedures taken up after the referral were performed under sufficient tooth isolation with a rubber dam.

**Figure 1:**
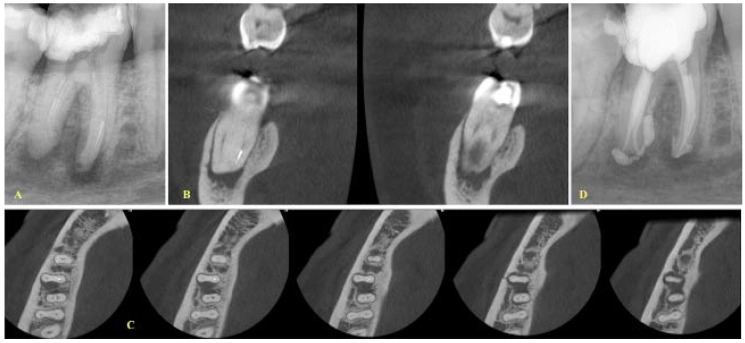
(**A**) Initial radiograph (mesial angle view) of a mandibular first molar with periapical lesions and a fragment at the apical third of the mesial root. (**B**,**C**). Coronal and axial views demonstrating that the two canals are separate, and the fragment is located in the mesiolingual canal, (**D**) A treatment plan decision to bypass the fragment was made due to the presence of a large lesion, especially at the lingual aspect of the cortical bone. Final radiograph of the case where the bypass of the fragment is evident.

**Figure 2:**
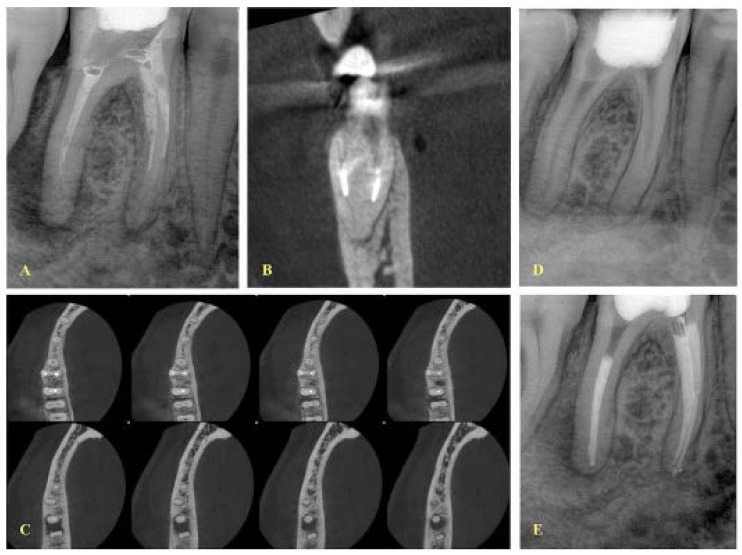
(**A**) Initial periapical radiograph (mesial angle view) of a mandibular first molar with periapical lesions and an instrument fracture at the middle third of the mesial root. The fragment is located at the mesiobuccal canal, according to the buccal-object rule. (**B**) CBCT coronal view indicating that the two canals are separate, (**C**) axial views demonstrating that the two canals are separate, however the two apical foramina are very close to each other, (**D**) radiograph after the removal of the fragment. (**E**) Final radiograph.

**Figure 3:**
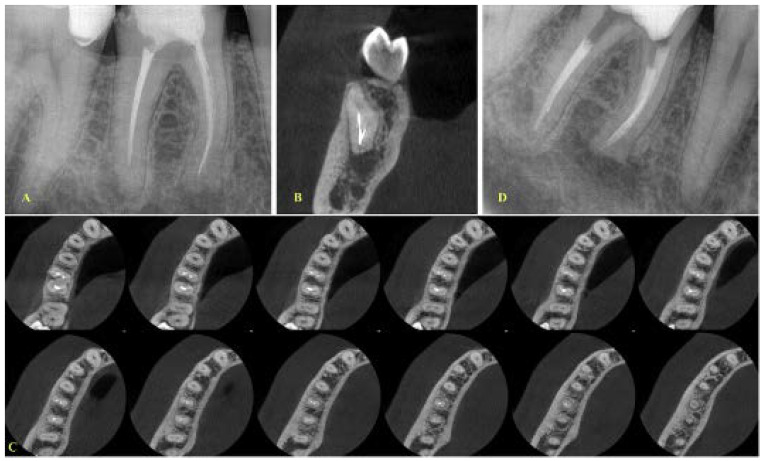
(**A**) Initial periapical radiograph of a mandibular first molar with periapical lesions and a fragment at the apical third of the mesial root (mesiobuccal canal), (**B**,**C**)**.** Coronal and axial views demonstrating that the two canals are merging at the apical third (**D**) Final radiograph with the fragment in place. No attempt was made to remove the fragment. Apical patency was achieved through the mesiolingual canal.

**Figure 4:**
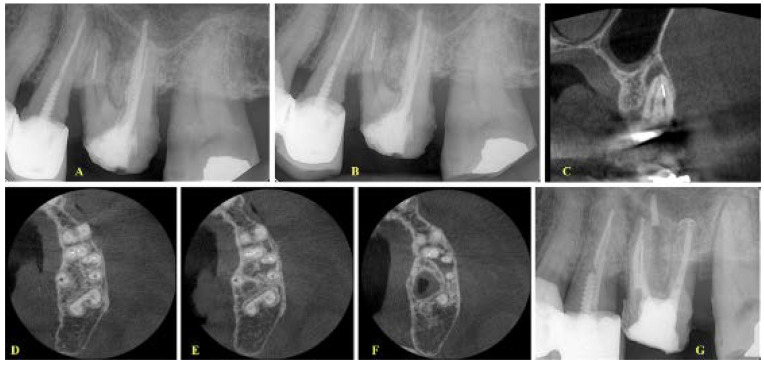
(**A**) Initial radiograph of a maxillary first molar with periapical lesions and an instrument fracture at the middle-apical third of the mesial root, (**B**) A second initial radiograph (mesial view) of the case, (**C**) Coronal view demonstrating that the fragment is located at the mesiobuccal 1 canal (MB1) and the two canals are merging at the apical third, (**D**–**F**) Consecutive axial views demonstrating that the two canals have a common apical foramen, (**G**) Final radiograph with the fragment in situ. No effort was made for the removal of the fragment. Apical patency was achieved through the mesiobuccal 2 canal (MB2).

**Figure 5:**
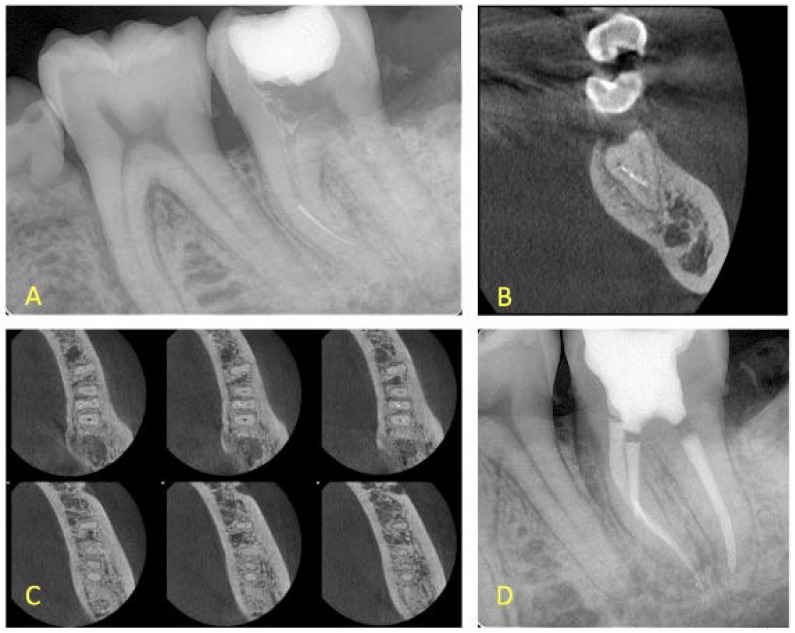
(**A**) Initial periapical radiograph of a mandibular second molar with an instrument fracture at the middle-apical third of the mesial root, (**B**,**C**) Coronal and axial views demonstrating that the fragment is located at the mesiolingual canal and the two canals are merging at the apical third, (**D**) Final radiograph in which the fragment has been removed with no excess dentin removal.

After the completion of the statistical part of the study and unmasking of the procedure, the evaluators were asked about the criteria used to set the treatment plan after CBCT examination. Briefly, they provided three different occasions as follows:

1. If a lesion was present, the canals were separate and the fragment was located at the apical level, and a final treatment plan of bypassing the fragment was set (Figure 1).
2. If the fragment was located at the middle level, the canals were separate and the curvature was not severe as indicated after evaluation of the coronal CBCT images; then, a removal attempt was made, especially in cases where the length of the fragment was short (Figure 2).
3. In cases where the canals were merging, then a final treatment plan of leaving the fragment in situ was set, irrespective of the presence or absence of lesions (Figure 3 and Figure 4). The only occasion that would induce a modification to this treatment plan (i.e., leave the fragment in situ) was to conclude, after CBCT assessment and evaluation of canal curvature, that the removal of the fragment located at the middle level of the root would not be of a high difficulty degree procedure (Figure 5).

The present study was performed using an observational paired design, which gives the advantage of comparing the same elements acquired by different radiographic assessments or evaluators in two different time periods. Nevertheless, the study was not free of limitations. No a priori sample calculation was performed, as this is the first study in the field; however, all of the patients who proceeded for treatment due to intracanal presence of fragments over a one-year period were included, revealing a considerably representative sample. In addition, the identified significant effects together with the uncertainty bounds recorded demonstrate that the study sample may be considered adequately powered to reveal these effects. In any case, presentation of the confidence intervals for the identified effect sizes allows for an estimation of the precision with regard to the treatment effect, indicating the grounds where the true effect is likely to appear in other similar studies [31,32]. Moreover, no blinding of the clinicians took place; however, this could not have been possible given the design of the study, and the fact that the clinicians already had prior knowledge of the case, from the initial examination with the periapical radiograph. However, statistical analysis was performed by an independent investigator who did not participate in any part of the examination of the patients and was blinded to data recordings for treatment planning through coding utilization.

In conclusion and so far, no clear recommendations and perspectives could be given about the clinical usefulness of CBCT in cases with fractured instruments. Three in vitro studies have been performed investigating a similar matter and supporting that periapical radiograph performed better than CBCT for the detection of fractured instruments located at the apical third of filled root canals [22,23,33]. This was also detected in the present study in some cases where filling materials were simultaneously present in root canals, along with the fragments. The present study highlights and justifies the necessity of prescribing a CBCT examination before any clinical action takes place in such high-difficulty cases. The current clinical proposal is in accordance with the conclusions of a detailed review by Venskutonis et al. [34], who supported that CBCT preoperative evaluation should be considered in cases where data obtained by periapical radiographs are not adequate to allow for an appropriate treatment planning and management. Nevertheless, the ALARA (as low as reasonably achievable) principle should always be considered upon the use of CBCT as a means for further diagnostic information in order to justify any additional radiation exposure. 

## 5. Conclusions

The present study reveals that CBCT preoperative evaluation may significantly aid with treatment planning and the management of cases with instrument fracture in mesial roots of mandibular and maxillary molars.

Apical location of the instrument and canal merging seem to influence the decision-making process in such cases.

## Figures and Tables

**Table 1:** Frequency distribution of characteristics of the sample according to whether there was a change or not in the initial treatment plan after CBCT evaluation for fractured instruments within the root canal (*n* = 52).

	Change in Treatment Planning						*p*-Value
	No		Yes		Total		
	N	%	N	%	N	%	
**Tooth (molar)**							0.25 *
*Mandibular (mesial root)*	14	38.9	22	61.1	36	100.0	
*Maxillary (mesiobuccal root)*	9	56.3	7	43.7	16	100.0	
**Canal location**							0.01 ^#^
*Mesiobuccal (mandibular)*	12	48.0	13	52.0	25	100.0	
*One Mesiobuccal (maxillary)* ^1^	9	69.2	4	30.8	13	100.0	
*Mesiobuccal & mesiolingual (mandibular)* ^2^	1	100.0	0	0.0	1	100.0	
*Mesiolingual (mandibular)*	1	10.0	9	90.0	10	100.0	
*Single canal (maxillary)* ^3^	0	0.0	3	100.0	3	100.0	
**Location (across root length)**							<0.01 ^#^
*Cervical*	2	100.0	0	0.0	2	100.0	
*Middle*	12	92.3	1	7.7	13	100.0	
*Middle-apical*	4	40.0	6	60.0	10	100.0	
*Apical*	5	18.5	22	81.5	27	100.0	
**Canal merge**							0.26 ^#^
*No*	11	52.4	10	47.6	21	100.0	
*Yes*	12	42.9	16	57.1	28	100.0	
*Single canal*	0	0.0	3	100.0	3	100.0	
**Presence of lesion**							0.71 ^#^
*No*	3	33.3	6	66.7	9	100.0	
*Yes*	20	46.5	23	53.5	43	100.0	
**Presence of symptoms**							0.63 *
*No*	5	38.5	8	61.5	13	100.0	
*Yes*	18	46.2	21	53.8	39	100.0	
**Total**	**23**	**44.2**	**29**	**55.8**	**52**	**100.0**	

CBCT, cone-beam computed tomography; * Pearson chi- square test; ^#^ Fisher’s exact test; ^1^ instrument fractured in one of two buccal canals in the mesial root of the maxillary molar; ^2^ instrument fractured in both buccal and lingual canals of the mesial root of the mandibular molar; ^3^ instrument fractured in the single canal of the mesiobuccal root of the maxillary molar.

**Table 2:** Frequency distribution of treatment planning decisions, after classic radiographic assessment compared to evaluation of CBCT of the same cases (*n* = 52 pairs).

		Conventional Periapical Radiography AssessmentN (%)			Total	*p*-Value *
		Retain Instrument w/o Bypass	Removal of Instrument	Bypass of Instrument		<0.001
**CBCT assessment** **N (%)**	**Retain instrument w/o bypass**	0 (0.0)	4 (16.0)	18 (66.7)	22 (42.3)	
	**Removal of instrument**	0 (0.0)	19 (76.0)	5 (18.5)	24 (46.2)	
	**Bypass of instrument**	0 (0.0)	2 (8.0)	4 (14.8)	6 (11.5)	
**Total**		0 (0.0)	25 (100.0)	27 (100.0)	52 (100.0)	

CBCT, cone beam computed tomography; * *p*-value for marginal homogeneity test for paired data (Stuart–Maxwell); w/o, without.

**Table 3:** Univariable and (plan for) multivariable logistic regression for the effect of tooth and jaw, location of breakage within the root canal (converted to binary: more cervically to apically), canal merge and presence of lesion or otherwise, on alteration of treatment plan from initial conventional periapical radiographic evaluation to final CBCT assessment.

	Univariable			Multivariable		
	Odds Ratio	95%CI	*p*-Value	Odds Ratio	95%CI	*p*-Value
**Tooth/jaw**			0.25			
*Mandibular*	Reference					
*Maxillary*	0.49	0.15, 1.63				
**Location of breakage**			<0.001			<0.001
*More cervically*	Reference			Reference		
*Apically*	11.31	3.07, 41.76		11.31	3.07, 41.76	
**Canal merge**			0.51			
*No*	Reference					
*Yes*	1.47	0.47, 4.57				
**Lesion**			0.47			
*No*	Reference					
*Yes*	0.58	0.13, 2.60				

CBCT, cone-beam computed tomography; Odds ratios (ORs) and 95% Confidence intervals are presented. (note: only one variable retained in the final model).

**Table 4:** Univariable and multivariable logistic regression for the effect of tooth and jaw, location of fracture within the root canal (converted to binary: more cervically to apically), canal merge and presence of lesion or otherwise, on treatment decision to remove or bypass versus retain the fractured instrument within the root canal, based on assessment through CBCT.

	Univariable			Multivariable		
	Odds Ratio	95%CI	*p*-Value	Odds Ratio	95%CI	*p*-Value
**Tooth/jaw**			0.02			0.05
*Mandibular*	Reference			Reference		
*Maxillary*	6.88	1.33, 35.58		7.26	0.99, 53.19	
**Location of breakage**			0.002			0.003
*More cervically*	Reference			Reference		
*Apically*	0.12	0.03, 0.45		0.03	0.003, 0.30	
**Canal merge**			0.01			0.01
*No*	Reference			Reference		
*Yes*	0.20	0.06, 0.71		0.05	0.01, 0.52	
**Lesion**			0.48			
*No*	Reference					
*Yes*	1.69	0.39, 7.26				

CBCT, cone-beam computed tomography; odds ratios (ORs) and 95% confidence intervals are presented.

## Data Availability

Available upon reasonable request to the corresponding author.
